# Laparoscopic Associating Liver Partition and Portal Vein Ligation for Staged Hepatectomy (L‑ALPPS) of Right Hemihepatectomy and Regional Lymph Node Dissection for Intrahepatic Cholangiocarcinoma (With Video)

**DOI:** 10.1245/s10434-025-17913-6

**Published:** 2025-08-23

**Authors:** Junyong Ma, Yizhou Wang, Yu Wang, Xifeng Li, Feng Shen, Xiaofeng Zhang

**Affiliations:** Department IV of Hepatic Surgery, Eastern Hepatobiliary Surgery Hospital, Navy Medical University, Shanghai, China

**Keywords:** L‑ALPPS, Intrahepatic cholangiocarcinoma, Laparoscopic right hemihepatectomy, Lymph node dissection, Minimally invasive surgery

## Abstract

**Background:**

Although the application of associating liver partition and portal vein ligation for staged hepatectomy (ALPPS) in intrahepatic cholangiocarcinoma (ICC) is still controversial, it continues to be attempted in selected patients. This video case discusses the key technical points for a complete laparoscopic ALPPS (L-ALPPS) of right hemihepatectomy and regional lymph node (LN) dissection for ICC.

**Methods:**

A 61-year-old ICC patient received L-ALPPS for right hemihepatectomy and regional LN dissection. The patient was deemed suitable as he only had two intrahepatic metastases, all in the right half of the liver, and no LN metastasis. The standardized remnant liver volume ratio (SRLVR) was only 38.17%, and the indocyanine green retention at 15 min (ICG-R15) was 11.9%.

**Results:**

The surgeries were carried out following the ‘easy-first’ and ‘no-touch’ principles. The operation time and bleeding volume were 300 min and 200 mL for stage I, respectively, and 140 min and 50 mL for stage II, respectively. For both stages, the patient was discharged 7 days after the surgery and had no postoperative complications. The histological grading of the tumor was T2N0M0. The patient received postoperative adjuvant chemotherapy, and all examinations showed no obvious abnormalities 5 months after surgery.

**Conclusions:**

L‑ALPPS provides an opportunity for radical surgery to selected ICC patients who have initially insufficient SRLVR and are intolerant to extensive hepatectomy. For these patients, by adhering to standardized laparoscopic techniques, surgeons can safely perform L‑ALPPS and regional LN dissection at resourceful laparoscopic surgery institutions.

**Supplementary Information:**

The online version contains supplementary material available at 10.1245/s10434-025-17913-6.

Associating liver partition and portal vein ligation for staged hepatectomy (ALPPS) is a two-stage surgical strategy that expands eligibility for curative liver resection by inducing rapid hypertrophy of the future liver remnant (FLR). It is primarily indicated for hepatocellular carcinoma (HCC) and colorectal liver metastases; however, evidence supporting ALPPS for other malignancies, such as intrahepatic cholangiocarcinoma (ICC), remains limited.^1–5^ Although ALPPS is now cautiously applied to ICC, reports remain largely limited to open approaches. This video illustrates key technical steps of laparoscopic ALPPS (L-ALPPS) for right hemihepatectomy with regional LN dissection in ICC.

## Methods

A 61-year-old male was found to have a 7.6 × 7.2 cm-sized mass with intrahepatic metastasis from abdominal magnetic resonance imaging (MRI) and positron emission tomography/computed tomography (PET/CT) scans. His serum carbohydrate antigen 19-9 (CA19-9) was 48.8 U/mL. The diagnosis was ICC without lymph node metastasis (LNM). Since the main tumor and the two intrahepatic metastases were all in the right half of the liver, there was an opportunity for radical surgery. While portal vein embolization (PVE) is a reliable and less invasive method for inducing FLR hypertrophy before right hemihepatectomy, a long time is needed, and in the presence of additional risk factors such as multifocal tumor, LNM, and progressive disease during hypertrophy, the overall survival is notably reduced.^6^ In contrast, ALPPS can induce rapid and extensive hypertrophy of the remnant liver volume, increase the resectability rate, and reduce the occurrence of postoperative complications.^7,8^

However, this patient belonged to the borderline resectable stage, and he required regional lymph node (LN) resection at the same time. The standardized remnant liver volume ratio (SRLVR) was only 38.17%, and the indocyanine green retention at 15 min (ICG-R15) was 11.9%. The probability of liver failure and other complications after direct surgical resection was relatively high, posing challenges to postoperative recovery and postoperative adjuvant therapy. After multidisciplinary consultations, we decided to perform L-ALPPS for right hemihepatectomy and regional LN dissection.

## Results

Both stages of L-ALPPS were performed laparoscopically. In stage I, the gallbladder and one of the intrahepatic metastases adjacent to the gallbladder were resected routinely. Histologically confirmed LNM is a crucial prognostic factor in ICC. For our patient, intraoperative rapid pathologic examination suggested adenocarcinoma, and ICC was considered based on the patient’s medical history. The key to surgery is ensuring the quality and safety of LN dissection.^9–11^ The extent of LN dissection is clearly defined by the American Joint committee on Cancer (AJCC).^12^ In the present case, we decided to perform LN dissection in stage I to minimize the possibility of LN metastasis and reduce the surgical difficulty in stage II caused by postoperative adhesion. The regional LN dissection followed a bottom-up, easy-first approach. The sequential resection of LNs adjacent to the hepatoduodenal ligament (group 12), the pancreatic head (group 13), and the common hepatic artery (group 8) greatly simplified the operation process, saved operation time, and ensured the quality of the operation.

Once the right liver was fully mobilized, intraoperative ultrasonography was used to mark the main trunk of the middle hepatic vein (MHV). The right hepatic pedicle was elastically suspended along the Laennec membrane. The Pringle maneuver was applied intermittently during the parenchymal transection. Hepatic resection was performed from the caudal to the cranial side along the MHV after the right hepatic pedicle was ligated. An anti-adhesion solution was applied to the liver wound and operation area to reduce postoperative adhesion. The patient was discharged on the seventh day after surgery and experienced no postoperative complications.

Stage II was carried out 13 days later, at which point the SRLVR had increased to 46.71%. The right hepatic pedicle and the right hepatic vein (RHV) were divided using a stapler. The operation time and the bleeding volume were 300 min and 200 mL for stage I, respectively, and 140 min and 50 mL for stage II, respectively. The abdominal drainage tube was removed on the fourth postoperative day. The patient was discharged on the seventh day after surgery and experienced no postoperative complications. The final pathologic examination revealed three active ICC lesions that were poorly differentiated small bile duct-type cholangiocarcinomas with microvascular invasion (MVI) grade M2. We accomplished a radical ICC surgery with R0 resection. The resection margin was 0.3 cm, and all dissected LNs (0/8) were negative. The histological grading was T2N0M0 (stage II). The patient received postoperative adjuvant chemotherapy, and all examinations showed no obvious abnormalities 5 months after surgery.

## Discussion

Minimally invasive approaches, including laparoscopic liver resections, have been associated with benefits such as reduced blood loss, shorter hospital stays, and comparable oncologic outcomes when compared with open surgery. These advantages are especially pertinent in complex procedures such as ALPPS, where minimizing surgical trauma is crucial. Furthermore, regional lymphadenectomy, which is often performed concurrently, plays a significant role in accurate staging and may influence long-term outcomes in ICC patients. L-ALPPS remains a subject of debate in clinical practice due to its technical complexity, requirement for specialized expertise, relatively large trauma, and higher complication rates. In addition, compression or manipulation of the tumor during stage I may risk tumor-cell dissemination.^13,14^ However, with rigorous selection and standardized laparoscopic technique, L-ALPPS can be a safe and effective option for selected ICC patients in high-volume centers. Patient selection should prioritize those in good general health, typically with, at most, three tumors (although source-based thresholds vary) confined to one liver hemi-lobe, no vascular invasion into the bile duct or vena cava, and no extrahepatic or nodal metastases.

We have the following recommendations for L-ALPPS: (1) Surgeons using energy devices (e.g., ultrasonic scalpels, electrocautery hooks) must avoid thermal damage to arterial vascular sheaths; opening sheaths along the vessel axis is a good technique. Assistants should refrain from excessive traction or blind clamping of vessels during field exposure. (2) Hepatic arterial variations must be identified during LN dissection; vessels should never be clamped or divided without clear confirmation. (3) The ‘easy-first’ and ‘no-touch’ principles are essential; elastic slings should be used to suspend vessels/bile ducts to optimize exposure and ensure complete removal of LNs, nerve plexuses, and connective tissue. (4) Vessels requiring transection must be ligated or sutured at their origin. The round ligament may be used to wrap the hepatic artery to prevent postoperative aneurysms/bleeding. (5) When LNs fuse or involve vessels, dissection complexity increases; expendable vessels (e.g., gastric coronary vein, superior pancreaticoduodenal vein, gastroduodenal artery) may be divided en bloc with lymph tissue to improve exposure. (6) For pancreatic head LN dissection, Kocher’s maneuver provides full exposure to remove nodes while protecting pancreatic tissue and preventing leaks.

## Supplementary Information

Below is the link to the electronic supplementary material.

Supplementary file1 (MP4 1,95,423 KB)
